# A novel method for tracking nitrogen kinetics in vivo under hyperbaric conditions using radioactive nitrogen-13 gas and positron emission tomography

**DOI:** 10.1152/japplphysiol.00859.2023

**Published:** 2024-02-29

**Authors:** Edward T. Ashworth, Ryotaro Ogawa, Juliana Nguyen, Chloe Afif, Rui C. Sá, Kim Butts Pauly, David R. Vera, Peter Lindholm

**Affiliations:** ^1^Department of Emergency Medicine, University of California San Diego, La Jolla, California, United States; ^2^Department of Radiology, University of California San Diego, La Jolla, California, United States; ^3^Department of Medicine, University of California San Diego, La Jolla, California, United States; ^4^Department of Radiology, Stanford University, Stanford, California, United States

**Keywords:** diving, decompression sickness, hyperbaric medicine, radiolabeling

## Abstract

Decompression sickness (DCS) is caused by gaseous nitrogen dissolved in tissues forming bubbles during decompression. To date, no method exists to identify nitrogen within tissues, but with advances in positron-emission tomography (PET) technology, it may be possible to track gaseous radionuclides into tissues. We aimed to develop a method to track nitrogen movement in vivo and under hyperbaric pressure that could then be used to further our understanding of DCS using nitrogen-13 (^13^N_2_). A single anesthetized female Sprague–Dawley rat was exposed to 625 kPa, composed of air, isoflurane, and ^13^N_2_ for 10 min. The PET scanner recorded ^13^N_2_ during the hyperbaric exposure with energy windows of 250–750 keV. The PET showed an increase in ^13^N_2_ concentration in the lung, heart, and abdominal regions, which all reached a plateau after ∼4 min. This showed that it is possible to gain noninvasive in vivo measurements of nitrogen kinetics through the body while at hyperbaric pressures. Tissue samples showed radioactivity above background levels in the blood, brain, liver, femur, and thigh muscle when assessed using a γ counter. The method can be used to evaluate an array of challenges to our understanding of decompression physiology by quantifying nitrogen load through γ counts of ^13^N_2_, and signal intensity of the PET. Further development of the method will improve the specificity of the measured outcomes, and enable it to be used with larger mammals, including humans.

**NEW & NOTEWORTHY** This article describes a method for the in vivo quantification and tracking of nitrogen through the mammalian body whilst exposed to hyperbaric pressure. The method has the potential to further our understanding of decompression sickness, and quantitatively evaluate the effectiveness of both the treatment and prevention of decompression sickness.

## Graphical abstract

**Figure:**
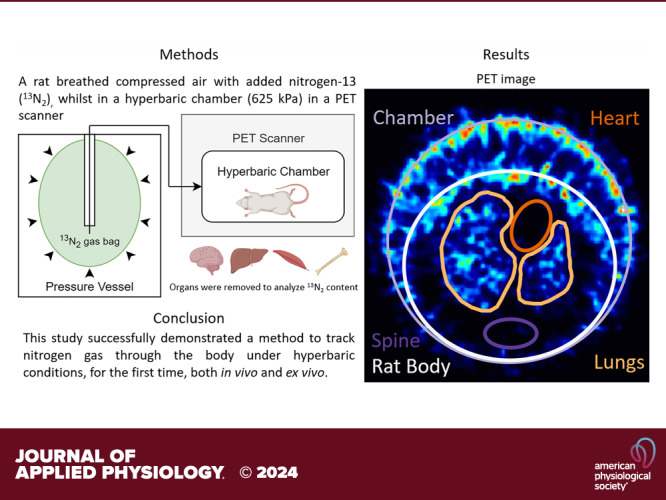

## INTRODUCTION

Decompression sickness (DCS) is an inherent risk of underwater diving, high-altitude aviation, and spaceflight that causes a range of symptoms including pain, organ dysfunction, coma, and death (1). The 17th century saw the first observations of DCS in animals, with humans first affected during pressurized mining operations in caissons in France reported during the building of the St. Louis and Brooklyn bridges (2, 3). Together, these operations observed that the rate of decompression influenced DCS risk; obese workers were more at risk, and recompression could improve symptoms (4). Paul Bert then confirmed these findings using animal models in a laboratory, showing that nitrogen was the gas causing the bubbles responsible for the observations in caisson workers, and provided recommendations for decompression rate to avoid bubble formation (3). John Haldane furthered these recommendations, providing the first dive tables for the Royal Navy following a series of experiments that evaluated symptoms in goats following differing decompression profiles (5). These experiments formed the idea of tissue half-times of saturation and desaturation that were dependent on organ fat content and blood flow. Haldane’s dive tables were adapted and used for the majority of the 20th century.

In 1976, ultrasound was used to detect venous gas bubbles for the first time (6), providing the first surrogate measurement of nitrogen content in vivo and a physiological measurement of DCS independent of reported symptoms. However, these measurements have been shown to correlate poorly with actual DCS symptoms and therefore cannot be relied upon (7). In lieu of good physiological measurements, computational modeling has been used to provide guidelines to avoid observed symptoms, with Paul Weathersby applying survival analysis to maximize the potential of the observations to prevent DCS (8). To date decompression theory is largely based on probabilistic and empirical evidence rather than experimental physiology (9). Despite 150 years of testing, there has been no physiological method for measuring nitrogen within the body under hyperbaric pressure.

Recently, we have used the radionuclide nitrogen-13 (^13^N_2_) to provide the first in vivo images of tissue nitrogen (10). As ^13^N_2_ decays, β-radiation is emitted with a half-life of ∼10 min. The emission can be imaged with positron-emission tomography (PET) to localize the radiation source, and therefore to localize the uptake of gaseous ^13^N_2_ in vivo (11). One previous study looked at the uptake of ^13^N_2_ gas in the human knee under normobaric conditions with a γ detector placed by the knee to track nitrogen kinetics (12). To date, we have only been able to examine the effects of ^13^N_2_ at normobaric pressure (10), or ex vivo after hyperbaric exposure (13). In addition, significant technological advances in PET imaging significantly improved spatial and temporal resolution while also using a much smaller radiation dose (14) than in the past. In this study, we develop an experimental model that could spatially and temporally track the movement of nitrogen through an intact mammal, during hyperbaric exposure.

## MATERIALS AND METHODS

**Figure 1.**
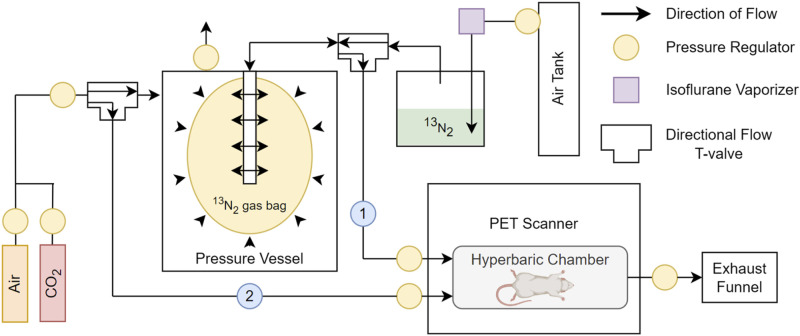
Schematic detail of the hyperbaric set up. The ^13^N_2_ gas is driven by air into the ^13^N_2_ gas bag, along with isoflurane. The pressure of delivered gas can be modulated by changing the pressure inside the pressure vessel to deliver ^13^N_2_ at hyperbaric pressures to the rat in the hyperbaric chamber (route 1) by changing the directional flow to allow flow back out of the gas bag, and into the hyperbaric chamber. Similarly changing the T-valve location allows CO_2_ to bypass the pressure vessel and euthanize the rat (route 2).

The ^13^N_2_ was created (PETNET, Siemens Medical Solutions, San Diego, CA) using a cyclotron in accordance with prior studies (15). Briefly, a liquid target containing aqueous NH_4_Cl solution (1.0 M, pH = 11) was irradiated with 15 MeV protons for 30 min.^13^N_2_ was extracted from the target by a helium sweep gas, which passed through a P_2_O_5_ absorber to purify the gas of NH_3_ and water vapor. The products were released into a vial, placed inside a lead ingot and casing, and delivered to the laboratory (transit time ∼10 min). The purity of ^13^N_2_ using this method has been shown to exceed 99.9% (15). Upon arrival at the laboratory, the ^13^N_2_ was placed into a dose calibrator and radioactivity was recorded. The vial was then shaken for 10 s and bubbled with air (to release the ^13^N_2_ gas from solution) at 1 L·min^−1^ directly from the isoflurane vaporizer (3% isoflurane; VS1482, Visual Sonics, Canada), into a balloon housed inside a pressure vessel (Fig. 1). This balloon contained a mix of air,^13^N_2_ and isoflurane (3%), that could be pressurized by the addition of external air within the pressure vessel (Fig. 1). The vial was then measured a second time to determine how much ^13^N_2_ had entered the gas bag (Fig. 1), whilst accounting for the decay of ^13^N_2_ (*Eq. 1*).

(*1*) $$A_{0}=\frac{A(t)}{e^{-\lambda \cdot t}}$$

*A*_0_ is baseline counts per minute, *A*(*t*) is counts per minute at the time point (*t*), and λ is equal to 0.693/9.965 min, where 0.693 is equal to the natural logarithm of 2, and 9.965 min is the half-life of ^13^N_2_.

A female Sprague–Dawley rat (8 mo, 324 g) was anesthetized using 5% isoflurane for ∼5 min. The rodent was inserted into a custom-built hyperbaric chamber (acrylic, 700 cm^3^, Hyperbaric Modular Systems, San Diego, CA), and the chamber was placed inside a stationary PET scanner (eXplore VISTA DR, GE Healthcare, Arlington Heights, IL). Imaging was centered on the thorax for a 10 min dynamic emission scan with energy windows of 250–750 keV, and set up to adjust for the decay rate of ^13^N_2_. The chamber was pressurized to an absolute pressure of 625 kPa (90.6 PSI, equivalent to 52 m of seawater) using the contents within the balloon, and immediately a 10 min PET scan was begun. A 10-min protocol was used due to uncertainties around the use of standard recommendations for rodent anesthesia at hyperbaric pressures, and to prevent carbon dioxide accumulation within the chamber that could affect physiological regulation. After the scan time elapsed, the chamber was flushed with carbon dioxide for 5 min to euthanize the rodent. The pressure was then rapidly released, and the animal was dissected. A mixed blood draw was taken from the heart, while the liver, brain, femur, and quadriceps muscle were surgically removed and placed into a γ counter (Gamma 8000, Beckman, IN) to obtain organ-specific counts, along with an empty vial to obtain a background measure that is subtracted from all samples. All organs were then weighed (CP64, Sartorius, Göttingen, Germany) to enable the calculation of counts relative to mass. All γ counts were obtained within a window of 400–600 keV and then corrected for the ^13^N_2_ half-life using *Eq. 1*. γ counts are reported as raw values, as well as normalized to the blood concentration to control for any variance, and the intrinsic unknown nature of the amount of ^13^N_2_ present in the breathed gas.

**Figure 2.**
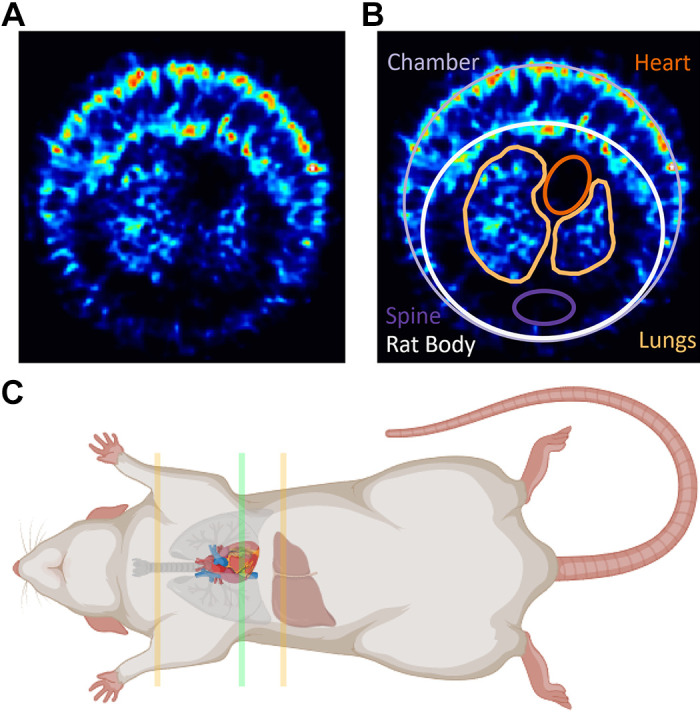
PET image of rat lung while breathing ^13^N_2_ gas under hyperbaric pressure (625 kPa) after 10 min. A raw image can be seen (*A*), as well as the image overlaid with anatomical features (*B*). The image shows a transverse view across the thorax (*C*, green window), clearly showing the lungs, from which an estimation of the heart can be obtained (*B*). The orange transverse windows (*C*) show the approximate range of the scanning window.

The 10-min scan was analyzed in five, 2-min segments. Each PET image recorded over 2 min was analyzed as a composite, nonoverlapping image using the Fiji image analysis software (16). An example image is shown in Fig. 2*A*. Images were converted into 3-D stacks, and regions of interest were drawn around four organs within the PET images. These were the lungs, the heart (estimated from the “gap” in between the lungs), the abdomen (estimated as the entire intraabdominal region below the level of the lungs), and the spinal cord (estimated as the space behind the lungs, in front of the shown in Fig. 2*B*). Mean and standard deviation (SD) of the signal intensity were calculated for each region of interest in each of the 2-min images.

The experiment was approved by The Institutional Animal Care and Use Committee Office at The University of California, San Diego (S19154).

## RESULTS

A baseline measurement of 339 MBq was recorded for the ^13^N_2_, which was subsequently bubbled, resulting in 100 MBq of ^13^N_2_ extracted into the balloon.

**Figure 3.**
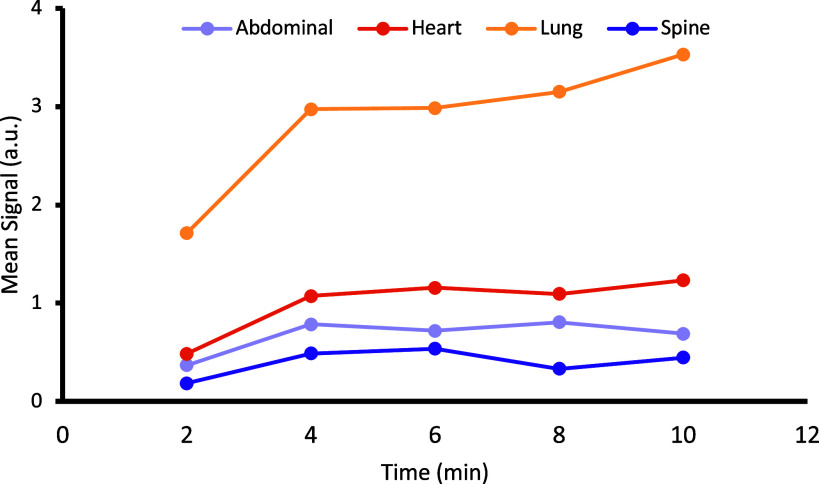
Signal intensity from different regions of interest in the rat in a PET scanner whilst breathing ^13^N_2_ at 625 kPa over a 10-min period, with signal taken as the mean of the prior 2 min. In all regions organs, there is a rapid increase over the first 4 min, with a flattening out of the signal over the following 6 min.

The signal in the four regions of interest showed a progressive increase in mean signal intensity over time, with a trend towards a plateau reached after 4 min (Fig. 3).

The euthanization of the rat took place in a 5 min CO_2_ exposure, with breathing stopping after 1 min 55 s. After the 5 min CO_2_ exposure, in 15 s the chamber was decompressed and the rat removed, with an additional 2 min 2 s taken for blood to be drawn from the heart. Within 2 min of the blood being drawn, all organs were removed and placed into the γ counter. Although the largest activity was seen in blood, all organs measured were substantially elevated above background radiation levels. The brain, muscle, and bone had notably low ^13^N_2_ counts.

## DISCUSSION

This study successfully demonstrates a method of tracking nitrogen gas through the body under hyperbaric conditions for the first time. Nitrogen imaging was achieved both in vivo using PET, and ex vivo using a γ counter to assess individual organs. The future use of this method in vivo, in hyperbaric conditions will allow quantitative assessment of DCS risk in mammals, enabling the evaluation of preventative and treatment strategies.

PET imaging showed ^13^N_2_ in the lungs, as expected (Fig. 2; Fig. 3). In all three analyzed areas the signal steadily rose, appearing to have begun to plateau around 4 min. The plateau suggests that the ^13^N_2_ exchange between the lungs and blood has reached an equilibrium. It would be expected that organ content would continue to increase past this time-point, but due to using PET without a colocalized anatomical reference (such as can be provided by MRI or CT), classifying individual organs was not possible without a significant spatial uncertainty. Indeed, it is possible that while an equalization occurred between the lungs and blood, the signal in each organ, is only the blood flow through that organ and not the amount of ^13^N_2_ present in the tissues themselves, as computational models predict longer half-times than that observed for nearly all tissues (8, 9). A longer exposure would help deduce whether a second plateau occurs later on that represents the equilibration of ^13^N_2_ in tissues.

In addition, the lack of colocalized anatomical references means that while the abdominal signal was likely primarily composed of signal from the liver, we are unable to infer to what extent due to this limitation. Likewise, signal from the region the spine is expected to be in is composed of connective tissue, muscle, bone, and the spinal column. When examining individual organs using such imaging modalities, using a larger animal, with larger organs, will likely assist with the resolution of ^13^N_2_ within those organs. Using a larger animal would require a more complex hyperbaric chamber design to ensure machine compatibility and animal safety, although the method presented herein is easily scalable for larger animals.

The 10-min time frame examined in the current study was relatively short to observe changes, but was required for ethical reasons, as the effects of anesthetics at depth were unknown. At this time, radioactive counts were still observed in most organs, despite no oxygen prebreathing, as shown previously. The minimal brain radioactivity (Table 1), suggests that there may be a protective mechanism against nitrogen entering into brain tissue, such as the blood-brain barrier. Furthermore, the spine region of interest had the lowest signal of each tissue assessed, which may suggest a similarly low uptake of ^13^N_2_ into the spinal cord, although further studies would need to confirm this. Tissues such as bone and muscle have been termed “slow tissues” due to their slow uptake of nitrogen under hyperbaric conditions (17). The short time frame may not have provided enough of a window for nitrogen uptake into these regions. Again, the use of PET alongside MRI or CT would enable organ-specific ^13^N_2_ count in vivo to be performed, allowing assessment of individual organ wash-in curves of ^13^N_2_, as in Fig. 3.

**Table 1.** Organ radioactivity counts following a 10-min exposure to ^13^N_2_ gas at 625 kPa hyperbaric pressure

Sample	Mass, g	Estimated Measurement Error, %	Counts·min^−1^·g^−1^	Counts Normalized to Blood, counts·min^−1^·g^−1^
Blood	3.85	1.02	1429.5	1.000
Liver	9.74	1.21	397.0	0.278
Brain	1.98	7.20	38.0	0.027
Muscle	2.03	5.29	83.5	0.058
Bone	0.53	7.66	118.6	0.083

The method and methodology first described here will further our understanding of decompression sickness, and enable a plethora of future studies that have a quantitative, physiological outcome variable, as well as allow for the study of the efficacy of different diving profiles and countermeasures in preventing DCS. Although this will not replace the current computational models, it will provide data to inform those models, rather than relying on DCS incidents to update the models. Furthermore, specific scenarios, such as in aviation or aerospace applications, can be assessed using this method, as well as treatment and preventative strategies.

Overall, we have described a method to successfully track nitrogen movement in vivo, under hyperbaric conditions. However, before this method can be applied properly, it needs improving to increase the scientific value obtained in each experiment. A better understanding of how anesthesia occurs at depth is required to facilitate prolonged exposures that better replicate actual dive times. This could be aided by an in-chamber monitoring system for animals, that would also aid with knowing when anesthesia has been completed, therefore accelerating the removal of the animal and subsequent γ counts of specific organs. In addition, future studies should look to use PET in combination with CT or MRI to allow for accurate spatial localization to enable organ-specific in vivo counts, while also evaluating experimental mechanisms to evaluate and quantify DCS risk under certain scenarios. Although this experimental paradigm has been devised for rodents, and will be further developed in rodents in the near future, it has the potential to be safely extended to larger animals and eventually humans.

## DATA AVAILABILITY

All data are available from the corresponding author upon reasonable request.

## GRANTS

This project was supported by a grant from the US Department of Defense; ONR grant number: N00014-20-1-276.

## DISCLOSURES

No conflicts of interest, financial or otherwise, are declared by the authors.

## AUTHOR CONTRIBUTIONS

E.T.A., R.C.S., K.B.P., D.R.V. and P.L. conceived and designed research; E.T.A., R.O., J.N., and C.A. performed experiments; E.T.A., R.O., J.N., C.A., and P.L. analyzed data; E.T.A., R.O., J.N., C.A., R.C.S., and P.L. interpreted results of experiments; E.T.A. prepared figures; E.T.A. drafted manuscript; E.T.A., R.C.S., K.B.P., and P.L. edited and revised manuscript; E.T.A., R.O., J.N., C.A., R.C.S., K.B.P., D.R.V., and P.L. approved final version of manuscript.
